# Impact of COVID‐19 Nonpharmaceutical Interventions on Respiratory Syncytial Virus Infections in Hospitalized Children

**DOI:** 10.1111/irv.13291

**Published:** 2024-04-23

**Authors:** Yingfeng Lu, Qinghui Chen, Shaolong Ren, Youyi Zhang, Liping Yi, Chen Qian, Jiaming Shen, Xiaofei Liu, Miao Jiang, Biying Wang, Jian Song, Xuejun Shao, Tao Zhang, Jianmei Tian, Genming Zhao

**Affiliations:** ^1^ Department of Epidemiology School of Public Health, Fudan University Shanghai China; ^2^ Department of Infectious Diseases Soochow University Affiliated Children's Hospital Suzhou China; ^3^ Clinical Laboratory Soochow University Affiliated Children's Hospital Suzhou China; ^4^ Children's Hospital of Fudan University, National Children's Medical Center Shanghai China

**Keywords:** acute lower respiratory infection, children, COVID‐19, respiratory syncytial virus

## Abstract

**Background:**

Nonpharmaceutical interventions (NPIs) targeted at SARS‐CoV‐2 have remarkably affected the circulation of other respiratory pathogens, including respiratory syncytial virus (RSV). This study aimed to assess the changes in epidemiological and clinical characteristics of RSV infections in hospitalized children before and during the pandemic in Suzhou, China.

**Methods:**

We prospectively enrolled children aged < 18 years who were hospitalized in Soochow University Affiliated Children's Hospital with acute lower respiratory infection (ALRIs) from January 2018 to July 2022. Changes in epidemiological and clinical characteristics of RSV infections were analyzed.

**Results:**

Compared with the same period in 2018–2019, the difference in the overall positive rate of RSV was not statistically significant in 2020, while it increased significantly in 2021 (11.8% [662/5621] vs. 20.8% [356/1711], *p* < 0.001) and 2022 (9.0% [308/3406] vs. 18.9% [129/684], *p* < 0.001). Specifically, the positive rates declined considerably from October to December 2020 but sharply increased during the summer of 2021. Compared to prepandemic period, RSV infections were more frequently observed in older children during the pandemic. RSV‐positive children exhibited milder clinical characteristics during the COVID‐19 pandemic, including decreased proportion of patients with hospital stay ≥ 11 days (10.3% vs. 6.7%, *p* < 0.05), less requirement for oxygen therapy (13.7% vs. 6.9%, *p* < 0.001), and fewer cases of polypnea (12.2% vs. 9.7%, *p* < 0.05) and wheeze (50.1% vs. 42.9%, *p* < 0.001).

**Conclusions:**

The implementation of multilayered NPIs targeted at COVID‐19 has affected the activity of RSV. Ongoing monitoring of RSV is warranted as the changing RSV epidemiology can provide valuable insights for future healthcare system planning.

## 1 Introduction

Respiratory syncytial virus (RSV) is one of the most common pathogens in children that cause acute lower respiratory infections (ALRIs), including bronchiolitis and pneumonia [1]. Globally in 2019, there were approximately 33 million RSV‐related ALRIs cases, 3.6 million hospitalizations, and over 100,000 deaths [2]. RSV poses a significant burden on healthcare systems as nearly all children are infected with RSV by 2 years of age, and the estimated RSV case‐fatality rate among children under 5 years of age is 6.21 per 1000 children [3].

The coronavirus disease 2019 (COVID‐19), caused by severe acute respiratory syndrome coronavirus 2 (SARS‐CoV‐2), rapidly spread at the end of 2019 and was declared a pandemic by the World Health Organization on March 11, 2020 [4]. To control the spread of SARS‐CoV‐2 in China, the government implemented comprehensive nonpharmaceutical interventions (NPIs), such as social distancing, improved hygiene regulations, mask‐wearing, stay‐at‐home orders, and school closures [5]. These public health measures, which caused unprecedented changes in society and healthcare systems, also impacted the activity of other viruses, one of which is RSV, as it shares similar routes of transmission with SARS‐CoV‐2.

Since the onset of the COVID‐19 pandemic, research regarding the impact of the pandemic on the circulating patterns of other respiratory pathogens has grown. Many regions had experienced a historically low RSV activity early in the pandemic followed by out‐of‐season resurgences [6, 7, 8]. Apart from the temporal trend of circulation, the changes in disease severity of RSV‐related ALRIs in children compared with the prepandemic period were observed in several studies as well [9, 10]. However, literature exploring the impact of COVID‐19 on temporal trends, as well as the disease severity of RSV, is limited in eastern China, and thus, further investigations are warranted as the changing epidemiological and clinical characteristics of RSV could impact future healthcare system planning.

Based on data from the only tertiary children's hospital in Suzhou, China, from 2018 to 2022, we aimed to assess the impact of the COVID‐19 pandemic on the epidemiological and clinical features of RSV infections among hospitalized children.

## 2 Methods

### 2.1 Study Site

The study was conducted in Suzhou, a major city in eastern China, with a population of approximately 12 million people. Suzhou consists of five municipal districts (Gusu, Wuzhong, Huqiu, Xiangcheng, and Industrial Park) and five county‐level cities. Soochow University Affiliated Children's Hospital (SCH) is the only tertiary children's hospital in Suzhou, with a ward complex capacity of around 1306 beds. According to our healthcare admission survey (HAS) conducted in 2011, the hospital admissions for all children in SCH accounted for 65.2% of the total hospital admissions in all medical institutions within five municipal districts in Suzhou [11]. It serves approximately 2.3 million outpatients and 70,000 inpatients annually [12].

### 2.2 Study Design and Participants

The present study enrolled children with ALRIs who were hospitalized at SCH between January 1, 2018, and July 30, 2022. The inclusion criteria for an ALRIs patient were the following: (1) Patients were aged younger than 18 years; (2) the discharge diagnosis contains International Classification of Diseases Version 10 (ICD‐10) disease codes J09–J18 (influenza and pneumonia) or J20–J22 (other ALRIs); (3) patients were not admitted to hospital for the same disease within 30 days prior to admission.

During the study period, Suzhou experienced two periods of strict implementation of NPIs, from February 2020 to April 2020 and from February 2022 to May 2022, respectively. The study had to be suspended for 2 months from February 2020 to March 2020 due to the COVID‐19 pandemic. The important events about the COVID‐19 pandemic in Suzhou, China, are detailed in Figure S1.

### 2.3 Data Collection

Data were collected through face‐to‐face questionnaire surveys conducted by trained investigators, medical record abstraction, and hospital information system information queries. The collected information included name, gender, date of birth, admission and discharge dates, underlying conditions such as congenital heart disease, clinical information (including clinical manifestations, laboratory findings, and treatment), and discharge diagnosis (ICD‐10 codes).

### 2.4 Specimen Collection and Testing

Nasopharyngeal secretions were collected within 24 h after admission from ALRIs children. RSV, adenovirus, and parainfluenza virus were detected using direct immunofluorescence assay (DFA), and influenza virus, rhinovirus, metapneumovirus, and bocavirus were detected using real‐time polymerase chain reaction (RT‐PCR). Sputum culture was used to detect respiratory bacterial pathogens including *Haemophilus influenzae* , *Streptococcus pneumoniae* , *Moraxella catarrhalis* , *Staphylococcus aureus* , and *Klebsiella pneumoniae* . Details regarding the respiratory pathogen testing are given in the Supporting Information.

### 2.5 Statistical Analysis

Categorical variables were described as number of cases and proportions (%). Chi‐squared tests or Fisher's exact tests were used to compare discrete variables. Non‐normally distributed continuous variables are expressed as medians and interquartile ranges (IQR) and compared using the Mann–Whitney–Wilcoxon test. Multivariable logistic regression models were developed to identify the potential factors associated with long hospital stay, incorporating all factors with *p* < 0.1 in the univariate analysis. Odds ratios (ORs) and 95% confidence intervals were reported. The Bonferroni correction was used to adjust test level for multiple comparisons, and the adjusted test level was α′ = 0.0167. Other tests were based on a two‐sided α with statistical significance defined as *p* < 0.05. All statistical analyses were performed using SAS, version 9.4 (SAS Institute Inc; Cary, North Carolina, USA).

## 3 Results

### 3.1 Characteristics of Study Population

During the study period, a total of 9016 patients who met the inclusion criteria was enrolled, including 3014 in 2018, 2607 in 2019, 1000 in 2020, 1711 in 2021, and 684 in 2022. There were 5432 males and 3584 females, for a male to female ratio of 1.52:1. The median age of patients in 2021 and 2022 was higher than in the same period in 2018–2019 (22 months vs. 13 months, 21 months vs. 12 months, *p* < 0.001). Most of patients were under 6 months old (2626, 29.1%), followed by the age group of 24–59 months old (2396, 26.7%) (Table 1).

**TABLE 1:** Basic characteristics of study subjects and detection of RSV, *n* (%).

	2018 (*n* = 3014)	2019 (*n* = 2607)	2020 (*n* = 1000)	*p* Value^a^ (2018–2019 vs. 2020)	2021 (*n* = 1711)	*p* Value^b^ (2018–2019 vs. 2021)	2022 (*n* = 684)	*p* Value^c^ (2018–2019 vs. 2022)	Total (*n* = 9016)
Gender									
Male	1882 (62.4)	1540 (59.1)	583 (58.3)	0.165	1004 (58.7)	0.103	423 (61.8)	0.673	5432 (60.3)
Female	1132 (37.6)	1067 (40.9)	417 (41.7)		707 (41.3)		261 (38.2)		3584 (39.8)
Age (month), *M* (IQR)	11 (3, 33)	17 (4, 47)	17 (5, 40)	0.180	22 (7, 45)	< 0.001	21 (5, 51)	< 0.001	16 (4, 42)
Age group (months)									
< 6	1068 (35.4)	763 (29.3)	251 (25.1)	< 0.001	360 (21.0)	< 0.001	184 (26.9)	< 0.001	2626 (29.1)
6–11	465 (15.4)	326 (12.5)	155 (15.5)	0.198	217 (12.7)	0.144	78 (11.4)	0.067	1241 (13.8)
12–23	519 (17.2)	404 (15.5)	183 (18.3)	0.184	304 (17.8)	0.191	99 (14.5)	0.140	1509 (16.7)
24–59	638 (21.2)	644 (24.7)	310 (31.0)	< 0.001	613 (35.8)	< 0.001	191 (27.9)	< 0.001	2396 (26.7)
≥ 60	324 (10.8)	470 (18.0)	101 (10.1)	< 0.001	217 (12.7)	0.130	132 (19.3)	< 0.001	1244 (13.8)
Detection of RSV	381 (12.6)	281 (10.8)	98 (9.8)	0.183	356 (20.8)	< 0.001	129 (18.9)	< 0.001	1245 (13.8)

*Note:* Figures are *M* (IQR) or numbers (%).

Abbreviations: COVID‐19, coronavirus disease 2019; *M* (IQR), median (interquartile range); RSV, respiratory syncytial virus.

^a^ Comparison between January and April to December of 2018–2019 and January and April to December of 2020.

^b^ Comparison between 2018–2019 and 2021.

^c^ Comparison between January to July of 2018–2019 and January to July of 2022.

### 3.2 Detection of RSV

From January 2018 to July 2022, the overall positive rate of RSV was 13.8% (1245/9016). Compared with the same period in previous prepandemic years (2018–2019), there was no significant difference in the positive rate of RSV in 2020 (11.3% [521/4630] vs. 9.8% [98/1000], *p* > 0.05), while it significantly increased in 2021 (11.8% [662/5621] vs. 20.8% [356/1711], *p* < 0.001) and 2022 (9.0% [308/3406] vs. 18.9% [129/684], *p* < 0.001) (Table 1).

**FIGURE 1:**
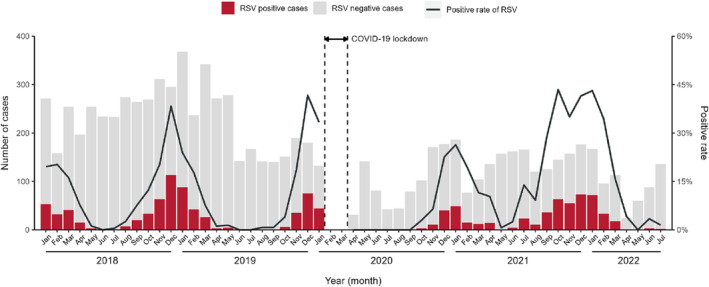
Monthly distribution of number of cases and positive rate of RSV among children from January 2018 to July 2022 in Suzhou, China. The area between the dotted lines represents the period of COVID‐19 lockdown.

We observed an impressive change in the seasonal pattern of RSV infections. As shown in Figure 1, before the COVID‐19 pandemic (2018–2019), the prevalence of RSV demonstrated a clear seasonal pattern in Suzhou, with cases more common from October to March and a low positive rate in summer months. However, there was a decline in RSV positive rates in 2020, particularly in November and December, which were significantly lower than the average levels of previous years (19.6% vs. 6.4% in November, 39.6% vs. 22.6% in December, *p* < 0.001). With the easing of NPIs, RSV activity returned to its usual level in early 2021. An off‐season RSV epidemic occurred during the summer of 2021, beginning in June 2021, and reaching a peak in October 2021. Additionally, the epidemic intensity of RSV was greater and lasted longer during 2021–2022 than before. With the exception of December 2021, the monthly positive rates of RSV in children with ALRIs from June 2021 to February 2022 were significantly higher than the average levels of previous years (*p* < 0.0167) (Table 2). Notably, during the pandemic, while there was a slight decrease in RSV cases, the reduction was relatively minor compared to non‐RSV cases (Figure 1).

**TABLE 2:** Month‐based comparison of RSV positive rates before and during the COVID‐19 pandemic, *n* (%).

Month	2018–2019	2020	*p* Value^a^	2021	*p* Value^b^	2022	*p* Value^c^
January	141/639 (22.1)	44/132 (33.3)	0.006	49/186 (26.3)	0.223	72/167 (43.1)	< 0.001
February	74/395 (18.7)	—	—	15/77 (19.5)	0.878	33/96 (34.4)	< 0.001
March	67/596 (11.2)	—	—	12/104 (11.5)	0.930	18/113 (15.9)	0.160
April	18/468 (3.9)	0/31 (0.0)	0.538	14/136 (10.3)	0.003	1/24 (4.2)	0.938
May	7/532 (1.3)	0/141 (0.0)	0.367	1/157 (0.6)	0.784	0/60 (0.0)	1.000^d^
June	0/376 (0.0)	0/79 (0.0)	—^e^	4/162 (2.5)	0.012	3/88 (3.4)	0.007^d^
July	1/400 (0.3)	0/42 (0.0)	1.000^d^	23/166 (13.9)	< 0.001	2/136 (1.5)	0.160^d^
August	8/415 (1.9)	0/43 (0.0)	1.000^d^	11/120 (9.2)	< 0.001	—	—
September	21/404 (5.2)	0/79 (0.0)	0.077	36/125 (28.8)	< 0.001	—	—
October	39/420 (9.3)	3/102 (2.9)	0.035	63/145 (43.5)	< 0.001	—	—
November	98/501 (19.6)	11/171 (6.4)	< 0.001	55/157 (35.0)	< 0.001	—	—
December	188/475 (39.6)	40/177 (22.6)	< 0.001	73/176 (41.5)	0.661	—	—

*Note:* Data were expressed as the positive number/the total number (%). The “—” indicated no available data. The adjusted test level for multiple comparisons was α′ = 0.0167.

^a^ Comparison between 2018–2019 and 2020.

^b^ Comparison between 2018–2019 and 2021.

^c^ Comparison between 2018–2019 and 2022.

^d^ Fisher's exact test.

^e^ Sum of rows or columns is zero, and no chi‐square value is calculated.

### 3.3 Age Distribution

**FIGURE 2:**
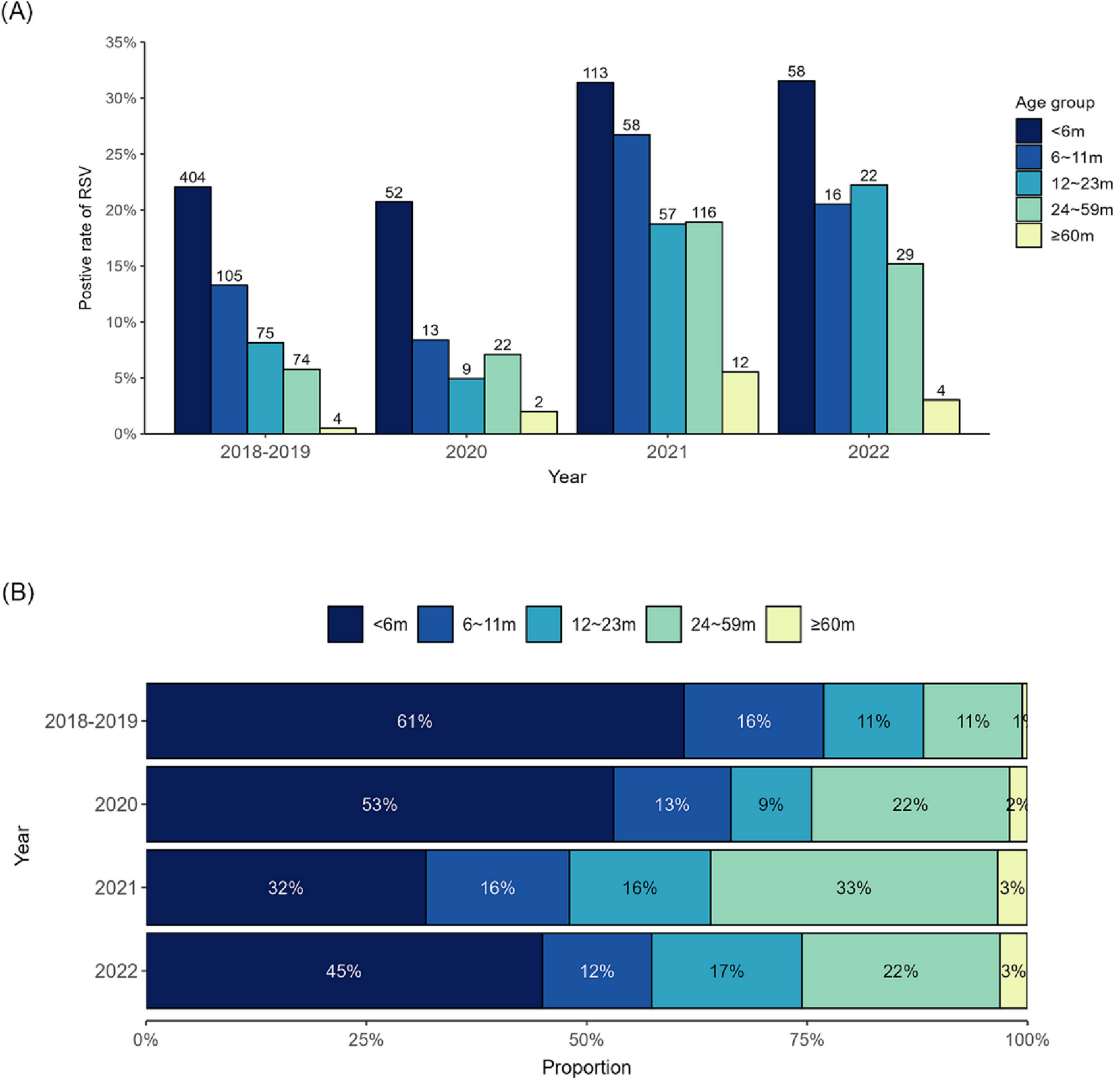
Age distribution of children positive for RSV, from 2018 to 2022. (A) Positive rates of RSV in children of different age groups. Bars represent positive rate of RSV. Number of RSV positive cases for each age group are listed above the bars. (B) The proportion of different age groups of RSV‐positive children.

The positive rate of RSV increased with a decrease in age. Children in the < 6‐month age group had the highest RSV‐positive rate. Compared with the pre‐COVID‐19 period (2018–2019), the positive rate of RSV across different age groups remained relatively stable in 2020 and increased significantly in 2021 and 2022 (*p* < 0.001). Children in the age group of 24–59 months and ≥ 60 months showed a more notable change compared to younger age groups, with their positive rates increasing from 5.8% and 0.5% in 2018–2019 to 18.9% and 5.5% in 2021–2022 (*p* < 0.001), respectively (Figure 2A and Table S1).

Among all RSV‐positive cases, children aged under 1 year of age account for the highest proportion. In the period of 2018–2019, 61.0% of RSV positive cases was less than 6 months of age. During the COVID‐19 pandemic, however, the proportion of cases under 6 months old decreased to a range of 31.7%–53.1%. Conversely, the proportion of positive cases in children over 1 year old has increased. The proportion of cases in children aged 24–59 months in 2021–2022 was significantly higher than in previous prepandemic years (*p* < 0.001), reaching a peak of 33% in 2021. Also, from before to during the COVID‐19 pandemic, the proportion of cases in children aged over 60 months has increased from 0.6% to 2.0%–3.4% (Figure 2B and Table S2).

### 3.4 Clinical Features of RSV‐Infected Children

The comparison of clinical characteristics of children with RSV infection before and during the COVID‐19 pandemic was shown in Table 3. Compared to the prepandemic period, there was a higher proportion of RSV‐positive children with fever symptoms (37.7% vs. 60.9%, *p* < 0.001), but there was a significant decrease in the proportion of children with polypnea (12.2% vs. 9.7%, *p* < 0.05) and wheeze (50.1% vs. 42.9%, *p* < 0.001). Although the median duration of hospitalization did not differ between two groups, during the COVID‐19 period, the third quartile of length of stay was shorter than before (7 days, IQR: 6–9 vs. 7 days, IQR: 6–8, *p* < 0.05), suggesting a decrease in the proportion of cases requiring longer hospitalization durations (Figure S2). To be noted, the proportion of patients with a length of stay ≥ 11 days significantly decreased during the pandemic (10.3% vs. 6.7%, *p* < 0.05). Additionally, a significantly smaller proportion of patients required oxygen therapy during the COVID‐19 period than before (13.7% vs. 6.9%, *p* < 0.001). The laboratory findings showed that the proportion of children with low WBC counts increased during the COVID‐19 pandemic (6.4% vs. 12.1%, *p* < 0.001).

**TABLE 3:** Clinical characteristics of RSV‐positive patients before and during the COVID‐19 pandemic, *n* (%).

Characteristics	Total (*n* = 1245)	Before COVID‐19 (*n* = 706)	During COVID‐19 (*n* = 539)	*p* Value^a^
Symptoms				
Fever	594 (47.7)	266 (37.7)	328 (60.9)	< 0.001
Cough	1242 (99.8)	706 (100.0)	536 (99.4)	0.161
Sore throat	1233 (99.0)	699 (99.0)	534 (99.1)	0.909
Polypnea	152 (12.2)	100 (14.2)	52 (9.7)	0.016
Dyspnea	11 (0.9)	9 (1.3)	2 (0.37)	0.167
Wheeze	624 (50.1)	393 (55.7)	231 (42.9)	< 0.001
Clinical course and treatment				
Length of stay (days), *M* (IQR)	7 (6, 9)	7 (6, 9)	7 (6, 8)	0.023
< 6	154 (12.4)	80 (11.3)	74 (13.7)	0.202
6–10	982 (78.9)	553 (78.3)	429 (79.6)	0.589
≥ 11	109 (8.8)	73 (10.3)	36 (6.7)	0.024
Oxygen therapy	171 (13.7)	134 (19.0)	37 (6.9)	< 0.001
Laboratory tests^b^ ^,^ ^c^				
WBC count > 12 (*10^9^/L)	231 (18.6)	124 (17.6)	107 (19.9)	0.310
WBC count < 5 (*10^9^/L)	110 (8.4)	45 (6.4)	65 (12.1)	< 0.001
CRP > 10 (mg/L)	199 (16.1)	101 (14.5)	98 (18.2)	0.073
Underlying conditions				
Any condition (≥ 1 condition)	63 (5.1)	24 (3.4)	39 (7.2)	0.002
Congenital heart defects	56 (4.5)	20 (2.8)	36 (6.7)	—
Chronic lung diseases	3 (0.2)	3 (0.4)	0 (0.0)	—
Neuromuscular disorders	2 (0.2)	1 (0.1)	1 (0.2)	—
Others	3 (0.2)	1 (0.1)	2 (0.4)	—
Viral coinfection				
Any virus (≥ 1 virus)	160 (12.9)	88 (12.5)	72 (13.4)	0.641
Influenza virus	48 (3.9)	43 (6.1)	5 (0.9)	—
Adenovirus	4 (0.3)	2 (0.3)	2 (0.4)	—
Parainfluenza virus	9 (0.7)	0 (0.0)	9 (1.7)	—
Rhinovirus	64 (5.1)	18 (2.6)	46 (8.5)	—
Bocavirus	37 (2.2)	25 (3.5)	12 (2.2)	—
Metapneumovirus	6 (0.5)	2 (0.3)	4 (0.7)	—
Bacterial coinfection				
Any bacteria (≥ 1 bacteria)	316 (25.4)	213 (30.2)	103 (19.1)	< 0.001
*Haemophilus influenzae*	99 (8.0)	78 (11.1)	21 (3.9)	—
*Streptococcus pneumoniae*	145 (11.7)	78 (11.1)	67 (12.4)	—
*Moraxella catarrhalis*	33 (2.7)	33 (4.7)	0 (0.0)	—
*Staphylococcus aureus*	73 (5.9)	54 (7.7)	19 (3.5)	—
*Klebsiella pneumoniae*	3 (0.2)	3 (0.4)	0 (0.0)	—

*Note:* Before COVID‐19, January 2018 to January 2020; during COVID‐19, April 2020 to July 2022.

Abbreviations: CRP, C‐reactive protein; WBC, white blood cells.

^a^ Comparison between the patients before COVID‐19 and during COVID‐19.

^b^ Data have missing values. Calculated based on the actual number of patients.

^c^ Normal range: white blood cell, 5.0–12.0 × 10^9^/L; C‐reactive protein, ≤ 10 mg/L.

No significant differences in viral coinfections were observed between two groups, while the proportion of children with bacterial coinfections decreased during the COVID‐19 period (30.2% vs. 19.1%, *p* < 0.001). A greater proportion of RSV‐positive children had underlying conditions during the COVID‐19 period (3.1% vs. 7.2%, *p* < 0.05), with congenital heart disease being the most common.

In multivariate analysis adjusted by before or during COVID‐19, we found that the odds of length of stay ≥ 11 days increased among children with underlying conditions, while age 12–23 months was significantly less likely to be associated with long hospital duration (Table S3).

## 4 Discussion

The present study demonstrates the alterations in the epidemic pattern and clinical features of RSV infection before and during the COVID‐19 pandemic. We observed a reduction in RSV activity in 2020, while an atypical surge of activity occurred during the summer of 2021, with a higher intensity compared to previous prepandemic years. Additionally, during the pandemic, RSV infections were more frequent in older children, and cases were clinically milder.

Unlike previous years where Suzhou RSV epidemics began in October, the activity of RSV was reduced in 2020, until an interseasonal spike in RSV in July 2021. The shift of the seasonality and intensity of RSV infections coincides with the implementation and subsequent relaxation of NPIs aimed at reducing the transmission of SARS‐CoV‐2 in the community. Our findings align with reports from other countries that have reported interseasonal RSV resurgence following a winter with reduced RSV activity during the COVID‐19 pandemic, including Germany, Australia, and Japan [6, 7, 13]. In England, an unprecedented summer surge of RSV activity occurred in 2021, including 11.6% higher test positivity [14]. Additionally, our results were similar to the surveillance data from 314 sentinel hospitals across mainland China between 2012 and 2021 [8]. Given that the transmission mechanisms of SARS‐CoV‐2 and RSV are the same, the reduction of RSV activity in 2020 is not unexpected in the context of NPIs [15]. Social distancing, stay‐at‐home orders, school closures, hygiene regulations, and especially mask‐wearing all have played a significant role in the suppression of RSV transmission. Of note, the atypical inter‐seasonal resurgence of RSV in summer 2021 may be explained by the concept of “immunity debt,” which suggests that children had reduced exposure to various pathogens and lacked immune stimulation during the lockdown. This may have increased the population of immunologically naive children, resulting in the resurgence of RSV upon the lifting of restrictions [16]. Immunity debt can pose a threat to the intensity and timing of the RSV epidemic, as well as the subsequent healthcare pressures [17]. Furthermore, we found that the decrease in the number of RSV cases was minor compared to non‐RSV cases during the pandemic, along with a significant reduction in RSV‐influenza coinfections. This finding suggested that RSV infections was less affected compared to other respiratory infections, especially influenza. Similar results were observed in studies from Shanghai and Guangdong province of China [18, 19]. This observation could possibly be attributed to the ability of RSV to spread rapidly through direct contact and its ability to survive longer period outside a host [20]. Besides, children under the age of 3 were not recommended to wear masks, resulting their inability to fully benefit from measures such as wearing masks [21].

Before the COVID‐19 pandemic, the greatest burden of RSV infections was observed in children younger than 1 year. Nevertheless, compared to prepandemic period, RSV infections were more frequently observed in older children during the pandemic. We observed a rise in the positive rate of RSV among older children, with a concurrent increase in the proportion of older children among all RSV‐infected cases during the pandemic. A similar older age structure of RSV patients was observed in many other countries; for example, an RSV surveillance in England revealed that compared to pre–COVID‐19 winters, the relative burden of RSV infections in older children was greater, and activity peaked at higher levels in the summer of 2021 [9, 14, 22]. Studies from Shanghai and Henan provinces of China also showed similar results [19, 23]. This finding is possibly a result of accumulation of immunologically naive children who have not been exposed to natural RSV infection during the lockdown, and as the NPIs were relaxed, these children also slightly increased in age [24]. The changing age structure raises challenges for healthcare providers to manage varying manifestations of RSV in older age groups.

Furthermore, it is interesting to note that the clinical phenotype of RSV positive children was milder during the COVID‐19 pandemic compared to previous years, as evidenced by a decreased proportion of children with longer hospitalization durations, less frequent requirement of oxygen therapy, and a reduced proportion of children with polypnea and wheeze. This finding was consistent with the studies from France, Japan, and Australia, where the disease severity decreased during the pandemic, based on an examination of clinical outcomes such as length of stay and intensive care admissions [9, 10, 25]. In contrast, a brief report from the United States indicated that infants suffered from more severe RSV‐related diseases during the pandemic [26]. The older age structure of RSV infections during the pandemic may account for the milder illness, as a younger age was associated with higher severity of RSV infection [27]. Besides, the decline in bacterial coinfections could contribute to the decrease in disease severity, as studies have shown that bacterial coinfections may increase the severity of the illness [28]. Several studies indicated that *Haemophilus influenzae* dominated microbiome profiles in children have been associated with enhanced mucosal proinflammatory responses [28]. Furthermore, patients residing outside Suzhou might travel to downtown Suzhou for better medical care when the illness became severe. However, the reduced mobility level during the pandemic potentially restricted non‐local patients with severe symptoms from traveling to Suzhou. Neither the frequency of viral coinfections nor underlying conditions could explain this milder clinical presentation during the pandemic, as these factors were observed either the same or more frequent than previous pre‐pandemic years. In addition, during the COVID‐19 pandemic in China, all children were required to undertake SARS‐CoV‐2 PCR tests upon seeking medical care at a hospital. Those who tested positive were isolated in designated medical facilities, while those who tested negative, regardless of the disease severity, continued to receive treatment at the hospital. Cases were not suspected to be COVID‐19 patients and sent elsewhere for treatment simply because they were more severe. Therefore, the severity of illness during the pandemic might not be affected in this aspect. A better understanding of how disrupted RSV transmission impacts the disease severity requires ongoing research.

This study has several limitations. First, the healthcare‐seeking behavior of patients may have been significantly altered during the COVID‐19 pandemic [29]. The observed decrease in patient numbers in this study may be attributed to reduced healthcare‐seeking behavior. For example, patients may choose online medical consultations or delay visits for fear of contracting COVID‐19 in a hospital setting. Besides, pandemic‐related restrictions on patient flow could limit access to healthcare services. Despite our results being derived from a test‐positive rate which is expected to be less sensitive to healthcare‐seeking behavior, it cannot be excluded that the pandemic has changed the profile of individuals who seek medical help for ALRIs. Second, while studies suggested good mask‐wearing compliance in China during COVID‐19, there was no compliance assessment regarding measures such as hand hygiene and maintaining social distance in Suzhou [30]. Third, the accuracy of the DFA test may have led to a slight underestimation of the RSV infection in this study. Fourth, the study was conducted at SCH, the only tertiary children's hospital in Suzhou, where 65.2% of all children residing in downtown Suzhou seek hospitalization [11]. While it did have some representativeness, being a single‐center study in Suzhou, the results might be different in other regions. Further investigations from other regions are needed to better understand the future epidemic pattern and clinical characteristics of RSV infections.

## 5 Conclusion

In conclusion, this study found that the RSV activity declined in 2020, whereas an atypical resurgence occurred during summer of 2021 in Suzhou. This phenomenon is likely caused by the introduction and subsequent relaxation of public health measures to mitigate the spread of SARS‐CoV‐2. Besides, during the pandemic, there has been an increase in RSV infections among older children, while the clinical phenotype of RSV was milder than before. These results suggested that more attention should be focused on RSV infections in older children. Despite the milder clinical symptoms observed during the pandemic, the burden of RSV disease continues to be significant. Ongoing monitoring of RSV is necessary to guide future healthcare system planning, as the scale and timing of future RSV epidemics remains to be seen.

## Author Contributions

**Yingfeng Lu:** Conceptualization; Data curation; Formal analysis; Software; Visualization; Writing – original draft. **Qinghui Chen:** Data curation; Project administration; Resources. **Shaolong Ren:** Data curation; Validation; Visualization. **Youyi Zhang:** Data curation; Validation. **Liping Yi:** Data curation; Validation. **Chen Qian:** Data curation; Validation. **Jiaming Shen:** Data curation. **Xiaofei Liu:** Data curation. **Miao Jiang:** Data curation. **Biying Wang:** Data curation. **Jian Song:** Data curation. **Xuejun Shao:** Data curation; Resources. **Tao Zhang:** Conceptualization; Methodology; Project administration; Supervision. **Jianmei Tian:** Conceptualization; Methodology; Project administration; Resources. **Genming Zhao:** Conceptualization; Methodology; Resources; Supervision; Writing – review and editing.

## Ethics Statement

The study was approved by the Institutional Review Board (IRB) of the School of Public Health, Fudan University.

## Conflicts of Interest

The authors declare no conflicts of interest.

### Peer Review

The peer review history for this article is available at https://www.webofscience.com/api/gateway/wos/peer‐review/10.1111/irv.13291.

## Supporting information


**Figure S1.** Important events of the COVID‐19 pandemic in Suzhou, China.
**Figure S2.** Comparison of the length of hospital stay (LOS) for RSV‐positive children before and during the pandemic. The central bar indicates the median LOS, and the lower and upper bounds of the box indicate the first and third quartiles (IQR). *P* value (Mann–Whitney–Wilcoxon test) comparing prepandemic and pandemic LOS is presented above the bars.
**Table S1.** Positive rate of RSV in different age groups before and during COVID‐19, *n* (%).
**Table S2.** The proportion of different age groups of RSV‐positive children before and during COVID‐19, *n* (%).
**Table S3.** Univariate and multivariate analysis of risk factors for length of stay ≥ 11 days in children infected with RSV.

## Data Availability

The data are not publicly available due to privacy or ethical restrictions. The data that support the findings of this study are available from the corresponding author upon reasonable request.
