# Supplementary material for: Impact of COVID‐19 Nonpharmaceutical Interventions on Respiratory Syncytial Virus Infections in Hospitalized Children
Source: Influenza Other Respir Viruses. 2024 Apr 23;18(4):e13291. doi: 10.1111/irv.13291 (PMC11039484; doi:10.1111/irv.13291)
Supplement: Supplementary file 1 — Figure S1. Important events of the COVID‐19 pandemic in Suzhou, China. Figure S2. Comparison of the length of hospital stay (LOS) for RSV‐positive children before and during the pandemic. The central bar indicates the median LOS, and the lower and upper bounds of the box indicate the first and third quartiles (IQR). P value (Mann–Whitney–Wilcoxon test) comparing prepandemic and pandemic LOS is presented above the bars. Table S1. Positive rate of RSV in different age groups before and during COVID‐19, n (%). Table S2. The proportion of different age groups of RSV‐positive children before and during COVID‐19, n (%). Table S3. Univariate and multivariate analysis of risk factors for length of stay ≥ 11 days in children infected with RSV. [file IRV-18-e13291-s001.docx]

**Supplementary Materials**

Impact of COVID-19 nonpharmaceutical interventions on respiratory syncytial virus infections in hospitalized children

**Supplementary Methods**

***Specimen collection***

Nasopharyngeal secretions were collected within 24 hours after admission from ALRIs children. This involved passing a suction catheter through the nose with the intent of passing it into the lower part of the pharynx. The depth of penetration for the nasopharyngeal aspirate catheter was set at 7-9 cm. A total of 2 mL nasopharyngeal aspirates were collected and sent to the laboratory at Soochow University Affiliated Children's Hospital (SCH) for pathogens detection within 30 minutes.

***Detection of viral pathogens***

RSV, adenovirus, and parainfluenza virus were detected using direct immunofluorescence assay (D^3^ Ultra DFA respiratory virus screening and identification kit, Athens, Ohio, USA). In brief, smears of exfoliated cells were air dried, which was followed by cold acetone fixation. Then, 10μl of monoclonal fluorescent antibodies was added to the smears. Specimens were incubated for 30 min in the dark at 37℃ and were then subject to a phosphate buffer saline (PBS, pH 7.4) wash. Slides were examined under a fluorescence microscope (excitation wavelength = 488 nm).

Total nucleic acid (DNA and RNA) was extracted from the collected specimen. Nucleic acid extracts were then tested for four viruses including influenza virus, rhinovirus, metapneumovirus, and bocavirus using the multiple respiratory pathogen panel assays (Health Gene Technologies, Ningbo, China). The primer/probes used and amplification condition were provided upon request. All steps of the nucleic acid extraction and real time-polymerase chain reaction (RT-PCR) test were conducted in parallel with positive and negative controls.

***Detection of bacterial pathogens***

The sputum specimens were plated on trypticase agar containing 5% sheep blood on chocolate agar, and incubated at 37°C for 18~24h in a 5% CO_2_ atmosphere. Isolated bacteria were primarily evaluated by colonial morphology, Gram staining, and were finally identified by biochemical reactions and API system. *Streptococcus pneumoniae* were differentiated by Optochin sensitivity. *Klebsiella pneumoniae* were identified by API 20E. *Haemophilus influenzae* and *Moraxella catarrhalis* were differentiated by API HN. *Staphylococcus aureus* were differentiated by API Staph.

**
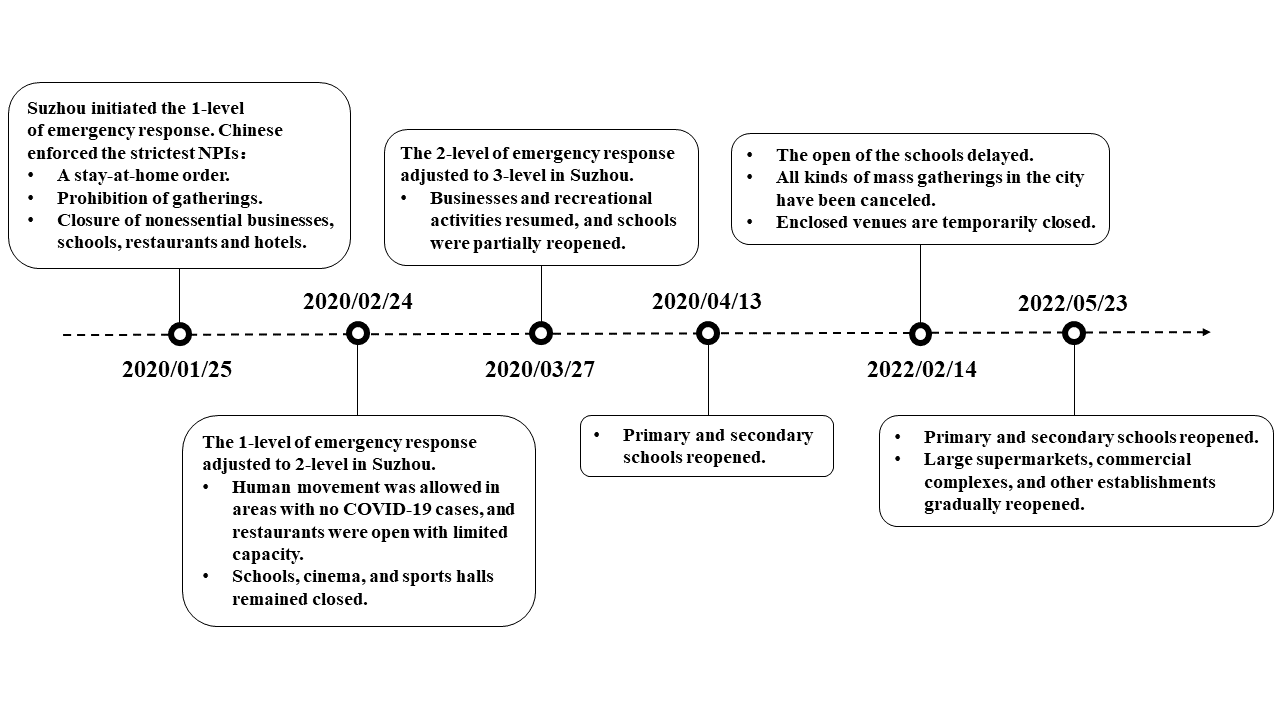
Supplementary Figures**

**Figure S1.** Important events of the COVID-19 pandemic in Suzhou, China.

**
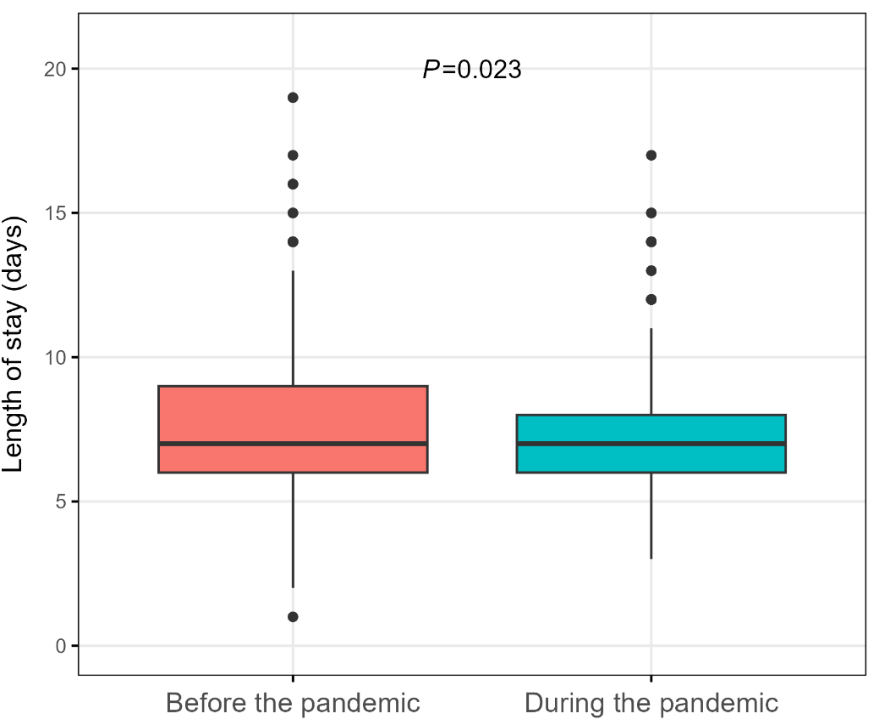
Figure S2.** Comparison of the length of hospital stay (LOS) for RSV-positive children before and during the pandemic. The central bar indicates the median LOS, the lower and upper bounds of the box indicate the first and third quartiles (IQR). *P* value (Mann-Whitney-Wilcoxon test) comparing pre-pandemic and pandemic LOS is presented above the bars.

**Supplementary Tables**

**Table S1.**  Positive rate of RSV in different age groups before and during COVID-19 [n (%)]

| Age group  (months) | 2018-2019 | 2020 | *P* Value^a^ | 2021 | *P* Value^b^ | 2022 | *P* Value^c^ |
| --- | --- | --- | --- | --- | --- | --- | --- |
| <6 | 404/1831  (22.1) | 52/251  (20.7) | 0.848 | 113/360 (31.4) | <0.001 | 58/184  (31.5) | <0.001 |
| 6~11 | 105/791  (13.3) | 13/155  (8.4) | 0.078 | 58/217  (26.7) | <0.001 | 16/78  (20.5) | 0.005 |
| 12~23 | 75/923  (8.1) | 9/183  (4.9) | 0.109 | 57/304  (18.8) | <0.001 | 22/99  (22.2) | <0.001 |
| 24~59 | 74/1282  (5.8) | 22/310  (7.1) | 0.345 | 116/613  (18.9) | <0.001 | 29/191  (15.2) | <0.001 |
| ≥60 | 4/794  (0.5) | 2/101  (2.0) | 0.126 | 12/217  (5.5) | <0.001 | 4/132  (3.0) | 0.048 |

^a^ Comparison between January, April to December of 2018-2019 with January, April to December of 2020.

^b^ Comparison between 2018-2019 with 2021.

^c^ Comparison between January to July of 2018-2019 with January to July of 2022.

**Table S2.**  The proportion of different age groups of RSV-positive children before and during COVID-19 [n (%)]

| Age group  (months) | 2018-2019  (n=662) | 2020  (n=98) | *P* Value^a^ | 2021  (n=356) | *P* Value^b^ | 2022  (n=129) | *P* Value^c^ |
| --- | --- | --- | --- | --- | --- | --- | --- |
| <6 | 404 (61.0) | 52 (53.1) | 0.344 | 113 (31.7) | <0.001 | 58 (45.0) | <0.001 |
| 6~11 | 105 (15.9) | 13 (13.3) | 0.385 | 58 (16.3) | 0.858 | 16 (12.4) | 0.858 |
| 12~23 | 75 (11.3) | 9 (9.2) | 0.366 | 57 (16.0) | 0.034 | 22 (17.1) | 0.043 |
| 24~59 | 74 (11.2) | 22 (22.5) | 0.004 | 116 (32.6) | <0.001 | 29 (22.5) | <0.001 |
| ≥60 | 4 (0.6) | 2 (2.0) | 0.532 | 12 (3.4) | <0.001 | 4 (3.1) | 0.233 |

^a^ Comparison between January, April to December of 2018-2019 with January, April to December of 2020.

^b^ Comparison between 2018-2019 with 2021.

^c^ Comparison between January to July of 2018-2019 with January to July of 2022.

| Variables | Univariate analysis | |  | Multivariate analysis | |
| --- | --- | --- | --- | --- | --- |
|  | OR (95% CI) | *P* value |  | aOR (95% CI) | *P* value |
| Before or During COVID-19 |  |  |  |  |  |
| Before | Reference |  |  | Reference |  |
| During | 0.62 (0.41-0.94) | 0.025 |  | 0.65 (0.43-1.04) | 0.070 |
| Gender |  |  |  |  |  |
| Male | Reference |  |  |  |  |
| Female | 0.90 (0.60-1.35) | 0.613 |  |  |  |
| Age group (months) |  |  |  |  |  |
| <6 | Reference |  |  | Reference |  |
| 6~11 | 1.25 (0.73-2.14) | 0.411 |  | 1.40 (0.81-2.41) | 0.234 |
| 12~23 | 0.19 (0.08-0.48) | <0.001 |  | 0.23 (0.09-0.59) | 0.002 |
| 24~59 | 1.41 (0.41-4.91) | 0.586 |  | 1.75 (0.49-6.22) | 0.388 |
| ≥60 | 0.93 (0.53-1.61) | 0.785 |  | 0.97 (0.56-1.69) | 0.915 |
| Viral co-infection |  |  |  |  |  |
| No | Reference |  |  |  |  |
| Yes | 1.49 (0.88-2.52) | 0.137 |  |  |  |
| Bacterial co-infection |  |  |  |  |  |
| No | Reference |  |  |  |  |
| Yes | 0.82 (0.51-1.31) | 0.340 |  |  |  |
| Underlying conditions |  |  |  |  |  |
| No | Reference |  |  | Reference |  |
| Yes | 2.34 (1.18-4.63) | 0.015 |  | 2.34 (1.18-4.63) | 0.018 |

**Table S3.** Univariate and multivariate analysis of risk factors for length of stay≥11 days in children infected with RSV

Abbreviations: OR, odds ratio; aOR, adjusted odds ratio; CI, confidence interval.
